# 

**Published:** 1893-12

**Authors:** 


					CONTENTS.
?dern Medical Journalism.
By J.
?Reig Smith, M.A., M.b'., C.m!
Aberd., F.R.S.E.
217
c0h, ?' *
"IPression of the Umbilical Cord
T uring Forceps Delivery.
By
Joseph
?LonH
1 uvpiJ I/GllIOt J?
Griffiths Swayne, M.D.
-ond.
229
A
of Double Empyema.
By
vy "? "UUUIO uiu[ijfciuai j->y
Djjm^Joknstqne Fyffe, B.A.,
233
? UUD. ...
,'hai'ases ?' Umbilical Hemorr-
tC?e.. occurring in the Same
?iy.
All 1115 111 1.11
By James Taylor,
* *-R-C.S. Eng.
eap?rf".Resorts? in the West of
rv?iv.and and South Wales.
phelten
enham.
By J. H. Garrett,
B.S. Dur., D.P.H. Camb.
241
Progress of the Medical Sciences:
Medicine.
By J. E. Shaw,- M.B.
233
Surgery.
By James Swain, M.D.
237
Ophthalmology.
By F. R. Cross
262
University College, Bristol. Faculty
of Medicine
266
Reviews of Books
270
Notes on Preparations for the
Sick ...
277
Meetings of Societies
281
The Library or the Bristol Medfco-
Chirurgical Society
282
Scraps
287

				

